# Supplementary material for: Prevalence of fungal infections in a patient cohort in The Gambia: Identification and characterization of three priority fungal species in patients with symptoms suggestive of TB
Source: PLoS One. 2026 Apr 24;21(4):e0347766. doi: 10.1371/journal.pone.0347766 (PMC13108815; doi:10.1371/journal.pone.0347766)
Supplement: Attachment — Submitted filename: Response to reviewers.docx [file pone.0347766.s003.docx]

Dear Dr. Singh and Reviewers,

We sincerely thank you for your thoughtful evaluation of our manuscript entitled “Prevalence of fungal infections in a patient cohort in The Gambia: Identification and characterization of three priority fungal species in patients with symptoms suggestive of TB” and for the constructive comments provided. We appreciate the opportunity to revise our work and have carefully addressed each point raised. Below, we provide a detailed, point-by-point response to the reviewers’ comments and indicate where changes have been made in the revised manuscript.

**Reviewer #1**

We thank the reviewer for the positive assessment of our manuscript and for the helpful suggestions to strengthen the work.

**Comment 1:**
*“Is there more information available on sociodemographic data? such as comorbidities, presence or not of cavitation in the chest xRay, socioeconomic data, housing conditions?”*

**Response:**
We agree that additional sociodemographic and clinical data would enhance the interpretation of our findings. Unfortunately, detailed socioeconomic, clinical and housing data were not systematically collected as part of the parent data sets that our samples derived from—so we were not able to include this in our analysis and demographics figures.

**Comment 2:**
*“What do you mean by TB status? Was TB confirmed for all samples? Were there ambulatory patients? hospitalized?”*

**Response:**
We appreciate this request for clarification. We have revised the manuscript to clearly define “TB status” as sputum positivity via GeneXpert (Page 4, Lines 124-125). We have also clarified that patients were recruited from a mix of ambulatory and inpatient settings, though the data set we utilized did not specify which category each patient fell into (Page 8, Lines 236-238).

**Comment 3:**
*The discussion could be extended on the public health implications of the multiplex PCR. With these results, what would be the application of this tool? and how should it be used, in which group could it be applied for a better target of population at risk? Authors state that low coinfection rate in may have been due to the small size of our study. However it seems that the samples tested might have had a low pre-test probability for fungal infections- What is the info/literature available on who to test?*

**Response:**
Thank you for this important suggestion. We believe that this has been partially addressed in (Page 3, Lines 69-74). We have included additional discussion on (Page 9, Lines 293-294). Regarding which group this test could be utilized in, we have included an additional statement in (Page 10, Lines 336-338).

We based our sample size off of several previous works:

For *Aspergillus sp.*, Lakoh et al demonstrated that 11.6% of patients presenting with respiratory symptoms had pulmonary aspergillosis in Sierra Leone. Granted, the HIV positivity rate in their sample population was ~50% which likely impacted the aspergillosis rate in comparison to our data set. This is discussed in (Page 10, Lines 303-305).

For *Histoplasma sp.,* Cornell et al had demonstrated a high seroprevalence in The Gambia (~30%). In Nigeria, Ekeng et al had demonstrated that in patients with a presumptive TB diagnosis, ~13% tested positive for pulmonary histoplasmosis. 35% of their patients were HIV positive. This is discussed in (Page 9, Lines 300-301).

For *Pneumocystis jirovecci,* Wills et al showed a >20% prevalence of PJP pneumonia in patients with HIV in this region. This is discussed in (Page 9, Lines 298-299).

Unfortunately, due to this being a secondary study utilizing samples derived from parent works, we were not able to include as many samples from HIV positive individuals as we had originally hoped. We agree that testing patients with HIV, as well as TB like symptoms, would’ve likely led to higher positive results. We discuss this in (Page 10, Lines 330-331).

**Reviewer #2**

We thank the reviewer for their positive feedback and constructive suggestions.

**Comment 1:**
*“Would have been great to include a large sample size.”*

**Response:**
We agree that a larger sample size would strengthen the study. We discussed this in our “Limitations” section. Future work will reflect clinical implementation of this assay and we hope that data derived from this will allow for consideration of a larger group of patients.

**Comment 2:**
*“What will be the cost of this multiplex assay in low-resource setting?”*

**Response:**
Thank you for highlighting this important aspect. We have included an approximate cost of $6.00 USD per sample in (Page 9, Lines 291-292)

**Additional Revisions and Journal Requirements**

- We have updated the Data Availability Statement to include the required third-party contact details.
- We have added the Role of the Funder statement to our cover letter
  “The funders had no role in study design, data collection and analysis, decision to publish, or preparation of the manuscript.”
- We have carefully reviewed and updated the reference list for completeness and accuracy.
- We have ensured that the manuscript complies with **PLOS ONE** formatting guidelines.
- We have completed and included the Inclusivity in Global Research questionnaire as Supporting Information.
- All changes in the manuscript are highlighted in the tracked changes version.

We believe that these revisions have significantly strengthened the manuscript and addressed all reviewer concerns. We are grateful for the reviewers’ insightful comments and the opportunity to improve our work.

Sincerely,
Dr. Zackary Salem-Bango and co-authors
